# Population–level effectiveness of PMTCT Option A on early mother–to–child (MTCT) transmission of HIV in South Africa: implications for eliminating MTCT

**DOI:** 10.7189/jogh.6.020405

**Published:** 2016-12

**Authors:** Ameena E Goga, Thu–Ha Dinh, Debra J Jackson, Carl J Lombard, Adrian Puren, Gayle Sherman, Vundli Ramokolo, Selamawit Woldesenbet, Tanya Doherty, Nobuntu Noveve, Vuyolwethu Magasana, Yagespari Singh, Trisha Ramraj, Sanjana Bhardwaj, Yogan Pillay, for the South Africa PMTCT Evaluation (SAPMCTE) Team

**Affiliations:** 1 Health Systems Research Unit, South African Medical Research Council, Cape Town, South Africa; 2 Department of Paediatrics, University of Pretoria, Pretoria, South Africa; 3 Centers for Disease Control and Prevention, Center for Global Health, Division of Global HIV and Tuberculosis, Atlanta, GA, USA; 4 School of Public Health, University of the Western Cape, Cape Town, South Africa; 5 UNICEF, New York, NY, USA; 6 Biostatistics Unit, South African Medical Research Council, Cape Town, South Africa; 7 School of Public Health and Family Medicine, University of Cape Town, Cape Town, South Africa; 8 Centre for HIV and STI, National Institute of Communicable Diseases, Johannesburg, South Africa; 9 Division of Virology and Communicable Diseases, School of Pathology, University of the Witwatersrand Medical School, Johannesburg, South Africa; 10 Department of Paediatrics and Child Health, Faculty of Health Sciences, University of Witwatersrand, Johannesburg, South Africa; 11 Wits School of Public Health, University of the Witwatersrand, Parktown, South Africa; 12 UNICEF, Pretoria, South Africa; 13 National Department of Health, Pretoria, South Africa

## Abstract

**Background:**

Eliminating mother–to–child transmission of HIV (EMTCT), defined as ≤50 infant HIV infections per 100 000 live births, is a global priority. Since 2011 policies to prevent mother–to–child transmission of HIV (PMTCT) shifted from maternal antiretroviral (ARV) treatment or prophylaxis contingent on CD4 cell count to lifelong maternal ARV treatment (cART). We sought to measure progress with early (4–8 weeks postpartum) MTCT prevention and elimination, 2011–2013, at national and sub–national levels in South Africa, a high antenatal HIV prevalence setting ( ≈ 29%), where early MTCT was 3.5% in 2010.

**Methods:**

Two surveys were conducted (August 2011–March 2012 and October 2012–May 2013), in 580 health facilities, randomly selected after two–stage probability proportional to size sampling of facilities (the primary sampling unit), to provide valid national and sub–national–(provincial)–level estimates. Data collectors interviewed caregivers of eligible infants, reviewed patient–held charts, and collected infant dried blood spots (iDBS). Confirmed positive HIV enzyme immunoassay (EIA) and positive total HIV nucleic acid polymerase chain reaction (PCR) indicated infant HIV exposure or infection, respectively. Weighted survey analysis was conducted for each survey and for the pooled data.

**Findings:**

National data from 10 106 and 9120 participants were analyzed (2011–12 and 2012–13 surveys respectively). Infant HIV exposure was 32.2% (95% confidence interval (CI) 30.7–33.6%), in 2011–12 and 33.1% (95% CI 31.8–34.4%), provincial range of 22.1–43.6% in 2012–13. MTCT was 2.7% (95% CI 2.1%–3.2%) in 2011–12 and 2.6% (95% CI 2.0–3.2%), provincial range of 1.9–5.4% in 2012–13. HIV–infected ARV–exposed mothers had significantly lower unadjusted early MTCT (2.0% [2011–12: 1.6–2.5%; 2012–13:1.5–2.6%]) compared to HIV–infected ARV–naive mothers [10.2% in 2011–12 (6.5–13.8%); 9.2% in 2012–13 (5.6–12.7%)]. Pooled analyses demonstrated significantly lower early MTCT among exclusive breastfeeding (EBF) mothers receiving >10 weeks ARV prophylaxis or cART compared with EBF and no ARVs: (2.2% [95% CI 1.25–3.09%] vs 12.2% [95% CI 4.7–19.6%], respectively); among HIV–infected ARV–exposed mothers, 24.9% (95% CI 23.5–26.3%) initiated cART during or before the first trimester, and their early MTCT was 1.2% (95% CI 0.6–1.7%). Extrapolating these data, assuming 32% EIA positivity and 2.6% or 1.2% MTCT, 832 and 384 infants per 100 000 live births were HIV infected, respectively.

**Conclusions:**

Although we demonstrate sustained national–level PMTCT impact in a high HIV prevalence setting, results are far–removed from EMTCT targets. Reducing maternal HIV prevalence and treating all maternal HIV infection early are critical for further progress.

Eliminating mother–to–child transmission of HIV (EMTCT) is pivotal to improving child survival in high HIV–burden, resource–limited settings [1]. South Africa, an archetypal high HIV prevalence, middle–income country, with social and political idiosyncrasies after an apartheid history, has prioritised EMTCT. Since 2014 this has been defined as <5% mother to child transmission of HIV (MTCT) at final endpoint in breastfeeding populations, ≤50 new infant HIV infections per 100 000 live births, ≥95% coverage of antenatal care among all women, ≥95% coverage of HIV testing and receipt of results and ≥90% coverage of antiretroviral drugs among HIV positive pregnant women [2,3]. Globally, strategies to prevent MTCT (PMTCT) are guided by a comprehensive four–prong approach, namely: (i) primary prevention of incident HIV infections; (ii) prevention of unplanned pregnancies; (iii) antiretroviral (ARV) drug interventions, and (iv) care, treatment and support, which aims to integrate PMTCT interventions into routine maternal, newborn and child health services [4].

Early, long–term triple combination antiretroviral therapy (cART) among HIV–positive women with higher CD4 cell counts (250–500 cells/mm^3^), or extended infant antiretroviral (ARV) prophylaxis have increased the impact of prong (iii) [5–7]. In 2010, the World Health Organization (WHO) PMTCT update recommended PMTCT Option “A” or “B” [8]: “A” provides antiretroviral prophylaxis (ARVP) from 14 weeks gestation for HIV–infected pregnant women with CD4 cell counts >350 cells/mm^3^ and infant nevirapine (NVP) prophylaxis throughout breastfeeding; or lifelong cART for HIV–infected pregnant women with CD4 cell counts ≤350 cells/mm^3^ or WHO stage 3–4 disease with 6 weeks of infant NVP prophylaxis; “B” provides cART during breastfeeding for all HIV–positive pregnant and lactating women with six weeks infant NVP or continued maternal cART beyond breastfeeding cessation if maternal CD4 cell count ≤350 cells/mm^3^or WHO stage 3–4 disease. Since 2013 a rapid shift to PMTCT Option B+ has occurred, and 18 of the 22 Global Plan priority countries (countries that house >90% of the world’s population of pregnant HIV positive women) have either endorsed, implemented or conducted national scale–up of PMTCT Option B+ [9]. “B+” has reduced final MTCT to <2% in non–breastfeeding countries [10].

South Africa’s national PMTCT program began with maternal and infant single dose NVP (sdNVP) in 2002, and transitioned to dual ARV therapy in February 2008, to WHO PMTCT Option A in April 2010, PMTCT Option B in April 2013 and PMTCT Option B+ in January 2015 [11].

Between 2001 and 2010, in resource–limited, high HIV prevalence countries, such as South Africa, rigorous routine measurements of national PMTCT impact and trends were simply unavailable. In 2010, using cross–sectional non–routine surveillance methodology at immunisation service delivery points we conducted the first national PMTCT effectiveness evaluation in South Africa, which documented a 3.5% (95% CI 2.9–4.1%) risk of MTCT, measured at 4–8 weeks postpartum (median 6 weeks), nationally under the 2008 PMTCT policy [12]. This paper presents the results of two subsequent national surveys, conducted to measure national and provincial–level PMTCT impact, measured 17–24 (August 2011–March 2012) and 31–38 (October 2012–May 2013) months after implementing PMTCT Option A, and during the first 2 months (April–May 2013) of transitioning to PMTCT Option B. During this time, the South African national PMTCT program aimed to reduce MTCT to less than 2% and less than 5% at six weeks and 18 months postpartum, respectively.

## METHODS

The methods have been explained in detail elsewhere [11,13]. In summary, two cross–sectional, facility–based, national epidemiological surveys were conducted between August 2011–March 2012 and October 2012–May 2013. Public health non–mobile facilities offering infant immunisation services in each of the nine provinces were stratified according to their six–week annual immunisation numbers and antenatal HIV prevalence [12,14]. Specifying relative precisions of 30% to 50% for the expected MTCT rate across provinces plus a design effect of 2 yielded a total desired sample size of 12 200 infant dried blood spot specimens (iDBS). Stratified two–stage sampling was used with facilities sampled with probability proportional size and with replacement [15]. At the second stage a fixed number of infants per facility, representing the median number of infants expected within the sampling window (three weeks in 8 provinces; four weeks in the sparsely populated, low HIV prevalence Northern Cape province), were sampled to ensure a self–weighting sample at provincial level. Data were gathered using a questionnaire adapted from several validated tools [13,16,17]. Trained study nurses recruited eligible consented infants (aged 4–8 completed weeks; receiving their six–week immunisation; not needing emergency care) and their caregivers [12]. Data on ARV exposure and infant feeding were self–reported [12]. Trained supervisors used standard operating procedures to monitor field work. Infants (not mothers) were tested for HIV antibodies to infant HIV exposure [11,13].

All infant dried blood spots (iDBS) were tested at the National Institute for Communicable Diseases, Johannesburg, using standardised accredited procedures, namely Enzyme immunoassay (EIA) (Genscreen HIV1/2 Ab EIA Version 2, Bio–Rad Laboratories, Schiltigheim, France) to detect HIV antibodies. All antibody–positive and 10% of negative specimens were re–tested using a second EIA (Vironostika HIV Uni–form II plus O, bioMérieux Clinical Diagnostics, Marcy–L’Etoile, France). Discordant results were re–processed using Western blot (GS HIV–1, Bio–Rad, Schiltigheim, France). iDBS with concordant positive or discordant EIA results or from self–reporting HIV–positive mothers were tested using a qualitative total nucleic acid Polymerase Chain Reaction (PCR) to determine infant’s HIV infection (COBAS AmpliPrep/COBAS TaqMan (CAP/CTM) Qualitative assay version 1.0, Roche Diagnostics, Branchburg NJ, USA).

For data analysis sample weights were calculated that consisted of two components: first realization weights were calculated depending on the realization within the strata of each province and second provincial weights. For the latter weight the number of live–births recorded across the nine provinces pertaining to the survey year was used. The sample weight was the product of realization weight and provincial weight and represents the number of live births the observed participant represents. The survey analysis took into account stratification, different sampling stages, the finite number of primary sampling units (PSU) and the design effect. Data were analyzed using SAS (SAS Institute N Carolina, Cary NC, USA) version 9.2and 9.4.

**Figure 1:**
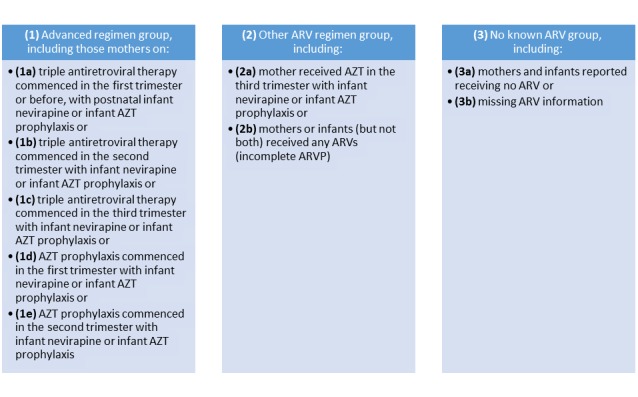
Classification of antiretroviral uptake 2011–2012 and 2012–13. Maternal self-reported antiretroviral uptake was classified into three overall groups with sub-categories in each group. AZT – azidothymidine, ARV – antiretroviral.

During data analysis self–reported maternal antiretroviral uptake was classified into three main groups with nine sub–groups (**Figure 1**).

Early MTCT by ARV category was initially measured using ARV definitions from **Figure 1**. Thereafter to facilitate comparison with the 2010 survey antiretroviral exposure was re–categorised into three main groups with six main categories, namely (i) “advanced regimen” group including mothers on cART or Azidothymidine (AZT) for >10 weeks and infant on sdNVP and/or AZT at birth (ARVP >10wks); (ii) “other ARV regimen” group including mothers on AZT for ≤10 weeks and infants on sdNVP and/or AZT at birth (ARVP ≤10 weeks) or mothers or infants (but not both) on any ARVs (incomplete ARVP) and (iii) “no known ARV group” including mothers and infants on no ARVs or with missing ARV information) [11].

Self–reported infant feeding was categorised using WHO definitions: exclusive breastfeeding (EBF), avoiding breastfeeding (FF) and mixed breastfeeding (MBF) [18].

Simple logistic regression followed by multivariable logistic regression was conducted to examine risk factors for early MTCT using pooled data from the 2011-12 and 2012-13 surveys.. Clinically important predictors and risk factors with *P* < 0.25 in univariate unweighted analysis were included in the preliminary main effects model. Variables with *P* < 0.05 or those that changed the odds ratio of the key exposure variable by 10% or those that were thought to be important independent predictors of MTCT in theoretical models were included in the penultimate model. The final model was selected based on model fit statistics (the best fit) with the lowest likelihood ratio and a significant model chi–square test (*P* ≤ 0.05).

Ethical approval was obtained from the Medical Research Council and the United States Centers for Disease Control and Prevention. All caregivers included in analyses provided informed consent.

## RESULTS

In the 2011–12 and 2012–13 surveys, respectively, 11 377 and 10 533 participants were screened and 10 482 and 9679 were enrolled; of the enrolled participants, iDBS were available on 10 106 (96.5%) and 9120 (94.2%) infants respectively, yielding a sample realization of 83% and 75% (**Table 1**). Provincial sample realization ranged from 73%–89% in 2011–12 and 56%–91% in 2012–13. The lower sample realization in 2012–13 is explained by the late (year–end) start date, vaccine stock–outs, facility changes from daily immunisation to weekly immunisation days and increased use of mobile services.

**Table 1:** Sample realization, infant HIV exposure and early MTCT at 6 weeks (range 4–8 weeks) postpartum: 2011–12 and 2012–13

**Province**	August 2011 – March 2012 survey*			October 2012 – May 2013 survey^†^		
**Sample realization number (%)**	**Weighted % Infant HIV–exposure**	**Weighted % MTCT (95% CI)**	**Sample realization number (%)**	**Weighted % Infant HIV–Exposure**	**Weighted% MTCT (95% CI)**
South Africa	10 106 (83%)	32.2 (30.7–33.6)	2.7 (2.1–3.2)	9120 (75%)	33.1 (31.8–34.4)	2.6 (2.0–3.2)
Eastern Cape	1194 (85%)	32.0 (29.6–35.5)	3.8 (2.1–5.5)	1035 (74%)	29.0 (25.1–32.9)	2.4 (1.1–3.8)
Free State	1056 (81%)	30.9 (28.6–33.3)	3.8 (2.3–5.3)	868 (67)	34.2 (30.6–37.7)	2.8 (1.5–4.1)‡
Gauteng	1607 (89%)	33.1 (29.8–36.4)	2.1 (0.9–3.4)	1637 (91%)	34.0 (30.6–37.4)	2.2 (1.3–3.1)
Kwa–Zulu Natal	1052 (75%)	44.4 (39.8–48.9)	2.1 (0.9–3.3)	1060 (76%)	43.6 (39.5–47.8)	2.9 (1.3–4.6)
Limpopo	1070 (76%)	23.0 (19.9–26.2)	3.1 (1.2–4.9)	1225 (88%)	25.2 (21.8–28.7)	2.1 (0.6–3.6)
Mpumalanga	1210 (76%)	35.6 (33.3–37.8)	3.3 (2.2–4.5)	898 (56%)	37.6 (33.6–41.7)	1.5 (0.6–2.3)‡
Northern Cape	506 (72%)	15.1 (12.7–17.5)	6.1 (2.5–9.6)‡	426 (61%)	20.9 (15.6–26.2)	2.2 (0.4–4.1)‡
North West	1037 (86%)	30.8 (28.5–33.1)	2.6 (1.1–4.0)	781 (65%)	31.4 (27.8–35.0)	5.4 (3.4–7.4)‡
Western Cape	1374 (98%)	17.8 (14.8–20.8)	2.0 (0.6–3.3)	1190 (85%)	22.1 (17.8–26.6)	1.9 (0.4–3.3)

MTCT – mother–to–child transmission, CI – confidence interval

*Conducted during months 17 (August 2011) to 24 (March 2012) after PMTCT Option A became policy.

**†**Conducted during months 31–38 after PMTCT Option A was adopted and includes the first 2 months (April and May 2013) of PMTCT Option B policy implementation.

‡Sample realization <70%, thus these MTCT estimates may be subject to bias.

### The weighted PMTCT cascade

**Figure 2:**
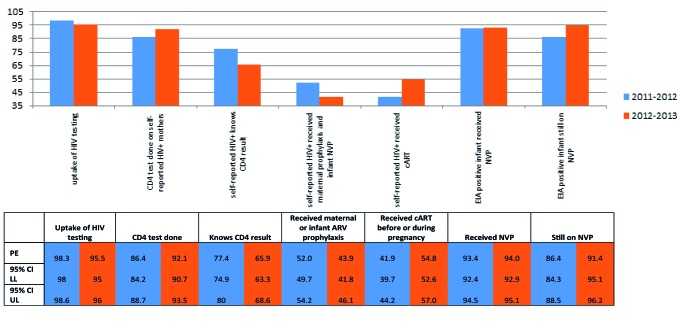
Weighted uptake along the PMTCT (prevent mother–to–child transmission) cascade among self–reported HIV positive women 2011–2012 and 2012–2013. PE – point estimate; LL – lower limit of 95% confidence interval (CI); UL – upper limit of 95% CI, cART – combination antiretroviral therapy, AZT – azidothymidine, ARV – antiretroviral, NVP – nevirapine.

Among all mothers self–reported weighted uptake of antenatal HIV testing was 98.3% (95% CI 98.0%–98.6%) in 2011–12 and 95.5% (95% CI 95.0–96.0%) in 2012–13 (**Figure 2**). In both surveys 99.4–99.8% of tested mothers received their results. This demonstrated that in 2012–13 almost 95% of all mothers were tested and received their HIV test results.

Among self–reported HIV–positive mothers 86.4% (95% CI 84.2–88.7%) and 92.1% (95% CI 90.7%–93.5%) reported being tested for CD4 cell count in 2011–12 and 2012–13, respectively, and 77.4% (95% CI 74.9–80%) and 65.9% (95% CI 63.3–68.6) reported knowing their CD4 results. The median reported CD4 cell count was 359 cells/mm^3^ (interquartile range IQR 240–499) in 2011–12 and 372 cells/mm^3^ (IQR 264–500) in 2012–13.

The proportion of self–reported HIV positive women receiving maternal cART increased by 12.9%, from 41.9% (95% CI 39.7–44.2%) in 2011–12 to 54.8% (95% CI 52.6%–57.0%) in 2012–13, while receipt of any maternal or infant prophylaxis dropped by 8.1%, from 52.0% (95% CI 49.7–54.2%) in 2011–12 to 43.9% (95% CI 41.8–46.1%) in 2012–13. Due to the increased cART uptake overall, ARV coverage among self–reported HIV positive women increased from 93.9% in 2011–12 to 98.7% in 2012–13.

Among EIA positive infants: 85.9% (95% CI 84.3–87.5) and 91.3% (95% CI 90.0–92.6%) were born to mothers who reported receiving any maternal antiretroviral therapy in 2011–12 and 2012–13 respectively; 93.4% (95% CI 92.4–94.5%) and 94.0% (95% CI 92.9–95.1%) reported initiating infant NVP prophylaxis in 2011–12 and 2012–13 respectively and 86.4% (95% CI 84.3–88.5%) and 91.4% (95% CI 95.1–96.2%) reported current infant NVP–use at the time of interview (six weeks postpartum); any breastfeeding increased from 53.0% (95% CI 50.4–55.5%) in 2011–12 to 72.2% (95% CI 70.1–74.2%) in 2012–13; EBF increased from 35.5% (95% CI 33.1–38.0%) in 2011–12 to 54.1% (95% CI 51.9%–56.2%) in 2012–13 and MBF increased from 14.0% (12.3–15.7%) in 2011–12 to 20.5% (18.8–22.1%) in 2012–13. Avoiding breastfeeding decreased from 47.1% (44.9–49.3%) in 2011–12 to 27.7% (95% CI 25.6–29.7%) in 2012–13.

### Weighted Infant HIV exposure and MTCT

In 2011–12 and 2012–13 self–reported maternal HIV positivity was 29.5% (95% CI 28.0–32.2%) and 32.1% (95% CI 30.8–33.4%) respectively while infant EIA positivity was 32.2% (95% CI 30.7–33.6%) and 33.1% (95% CI 31.8–34.4%) respectively (**Table 1**). The national population–level risk of early MTCT measured among EIA positive infants was 2.7% (95% CI 2.1–3.2%) in 2011–12 and 2.6% (95% CI 2.0–3.2%) in 2012–13. Early MTCT varied provincially from 2.0% (95% CI 0.6–3.3) to 6.1% (95% CI 2.5–9.6) in 2011–12 and from 1.5% (95% CI 0.6–2.3) to 5.4% (95% CI 3.4–7.4) in 2012–13 (**Table 1**). The unadjusted pooled early MTCT (2011–12 and 2012–13) was 2.6% (2.2–3.1%).

**Figure 3:**
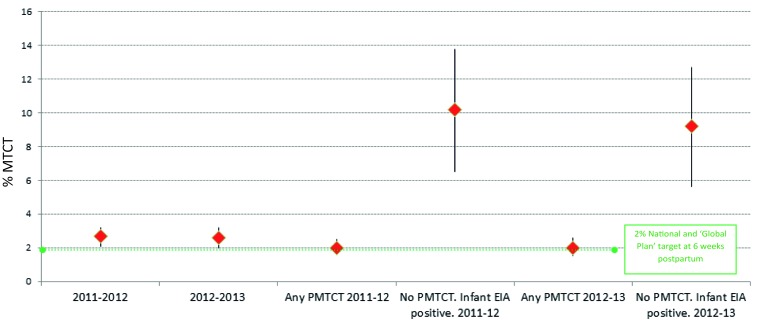
MTCT (mother–to–child transmission) measured in 2011–12 and 2012–13, with and without any PMTCT (prevent mother–to–child transmission) intervention. EIA – enzyme immunoassay

In 2011–2012 and 2012–13 among “mothers receiving any PMTCT intervention(s)”, early MTCT was 2.0%, (95% CI 1.6–2.5% and 1.5–2.6%, respectively); among mothers who did not know that their infants were EIA–positive, early MTCT was 10.2% in 2011–2012 (95% CI 6.5–13.8%) and 9.2% in 2012–2013 (95% CI 5.6–12.7%), respectively (**Figure 3****)**.Overall, the risk of unadjusted MTCT differed significantly by antiretroviral exposure (**Table 2**, *P* < 0.0001 for Columns A–C). Columns B and C present early MTCT using the pooled 2011–13 data set analyzed according to the 2010 survey ARV categories [11], to facilitate comparison. Of note is that early MTCT decreased to 1.2% (0.6–1.7) among HIV positive mothers who commenced cART in the first trimester or before.

**Table 2:** MTCT by various antiretroviral exposures: pooled 2011–12 and 2012–13 data

Antiretroviral regimen	Unweighted frequency of observations (weighted frequency)	Unweighted frequency of HIV positive infants (weighted HIV positive frequency)	Weighted MTCT (%) (Column A)	Weighted MTCT (%) according to ARV groups generated in the 2010 paper [11] (Column B)	Weighted MTCT (%) according to ARV groups generated in the 2010 paper [11] (Column C)
**Advanced regimen group:**					
(1a) cART commenced in 1st trimester or before with infant postnatal prophylaxis	1472 (187 986)	19 (2201)	1.17 (0.61–1.72)	**2010 1a,** 1.60 (1.15–2.05)	1.84 (1.44–2.25)
(1b) cART commenced in 2nd trimester with infant prophylaxis	727 (96 677)	14 (1638)	1.69 (0.76–2.62)		
(1c) cART commenced in 3rd trimester with infant prophylaxis	163 (35 699)	2 (330)	0.92 (0.00–2.22)		
(1d) AZT prophylaxis commenced in 1st trimester with infant prophylaxis (ARVP)	821 (102 640)	20 (2619)	2.54 (1.28–3.80)	**2010 1b,** 2.30 (1.42–3.2)	
(1e) AZT prophylaxis commenced in 2nd trimester with prophylaxis (ARVP)	1087 (151 221)	26 (3515)	2.31 (1.42–3.21)		
**Other ARV regimen group:**					
(2a) AZT prophylaxis commenced in 3rd trimester with prophylaxis (ARVP)	205 (27 856)	2 (368)	1.32 (0.00–3.16)	**2010 2a,** 1.32 (0.00–3.16)	2.49(1.36–3.61)
(2b) mothers or infants (but not both) received any ARVs (incomplete ARVP)	507 (62 419)	19 (2446)	3.90 (1.88–5.92)	**2010 2b,** 3.90 (1.88–5.92)	
**No known ARV group:**					
(3a) mothers and infants reported receiving no ARV	63 (8119)	8 (995)	**2010 3a,** 11.40 (3.75–19.05)		
(3b) missing ARV information	643 (76 929)	48 (5875)	**2010 3b,** 7.28 (5.03–9.53)		

MTCT – mother–to–child transmission, cART – combination antiretroviral therapy, AZT – azidothymidine, ARV – antiretroviral, ARVP – antiretroviral prophylaxis

**Table 3** illustrates that controlling for maternal age, socio–economic status (SES), marital status, education, gestational age at first ANC visit, total number of lifetime pregnancies, whether or not the current pregnancy was planned, province, survey year and whether or not the infant weighed less than 2.5kg at birth, the adjusted odds of early MTCT increased significantly if mother started ARVP in the second trimester (after 12 weeks’ gestation) or if only mother or baby or neither received antiretroviral drugs, compared to mothers who received cART in the first trimester or before. There were no additional significant MTCT differences between any of the other ARVP groups, although this could relate to small sample sizes. However the point estimates for early MTCT tended to increase as ARV exposure decreased.

**Table 3:** Associations between key PMTCT interventions and weighted perinatal infant HIV positive status in HIV exposed infants, South Africa, pooled data 2011–2013

Indicators	Frequency of HIV exposed infants with PCR results*, n = 5889 (Nw = 762 314)	Frequency of HIV infected infant n = 160, (Nw = 20 130)	Unadjusted OR, (95% CI)	Adjusted OR†, Model 1§	Adjusted OR†, Model 2||	Adjusted OR†, Model 3¶	Adjusted OR‡, final Model**
**CD4:**††							
≤350 cells/mm^3^	1682 (224 217)	35 (3977)	0.87 (0.51–1.50)	0.98 (0.42–2.05)			0.96 (0.46–2.01)
>350 cells/mm^3^	1927 (258 908)	35 (5258)	Ref	Ref			Ref
Missing	2230 (272 433)	90 (10 895)	2.01 (1.30–3.1)	0.79 (0.40–1.55)			0.78 (0.40–1.51)
**Advanced regimen group** (as defined in **Figure 1**):							
(1a)	1472 (187 986)	19 (2201)	Ref	Ref			Ref
(1b)	727 (96 677)	14 (1638)	1.45 (0.70–3.005)	1.84 (0.81–4.18)			1.82 (0.8–4.1)
(1c)	163 (35 699)	2 (330)	0.79 (0.18–3.46)	0.43 (0.05–3.56)			0.44 (0.05–3.56)
(1d)	821 (102 640)	20 (2619)	2.21 (1.08–4.52)	2.06 (0.78–5.44)			2.07 (0.79–5.43)
(1e)	1087 (151 221)	26 (3515)	2.01 (1.09–3.7)	2.43 (1.13–5.23)			2.37 (1.1–5.15)‡‡
**Other ARV regimen group** (as defined in **Figure 1**):							
(2a)	205 (27 856)	2 (368)	1.13 (0.25–5.06)	1.32 (0.25–6.83)			1.25 (0.24–6.39)
(2b)	507 (62419)	19 (2446)	3.44 (1.66–7.14)	4.12 (1.53–11.43)			4.16 (1.53–11.35)‡‡
**No known ARV group** (as defined in **Figure 1**):							
(3a)	63 (8119)	8 (995)	11.78 (4.83–28.75)	9.04 (2.14–38.19)			9.0 (2.17–37.38)‡‡‖
(3b)	643 (76 929)	48 (5875)	7.0 (3.85–12.65)	11.90 (4.64–30.49)			11.58 (4.46–30.05)‡‡
**Infant feeding practices:**							
FF	2212 (289 615)	46 (6218)	Ref		Ref		Ref
EBF	1452 (192 577)	41 (6044)	1.77 (1.09–2.87)		1.98 (1.08–3.66)		1.80 (0.95–3.42)
Mixed BF	365 (46 619)	12 (1379)	1.67 (0.88–3.12)		1.48 (0.76–2.88)		1.04 (0.52–2.10)
**Delivery type:**							
Cesarean	1322 (178 656)	31 (4385)	Ref			Ref	Ref
Vaginal	4404 (563 637)	124 (15234)	0.91 (0.58–1.42)			0.46 (0.20–1.05)	0.82 (0.43–1.59)

PMTCT – prevent mother–to–child transmission, EBF – exclusive breastfeeding, FF – formula feeding (no breastmilk), MF – breastfeeding with other fluids or solids, ARV – antiretroviral, CI – confidence interval, OR – odds ratio, Nw – weighted population number, Ref – reference group

*55 (unweighted) exposed infants with missing HIV DNA test results excluded from this analysis.

†Adjusted for maternal age, SES, marital status, education, gestational age at first ANC visit, total number of lifetime pregnancies, whether or not the current pregnancy was planned, province, survey year and whether or not the infant weighed less than 2∙5kg at birth.

‡Adjusted for maternal age, SES, marital status, education, gestational age at first ANC visit, total number of lifetime pregnancies, whether or not the current pregnancy was planned, province, survey year and whether or not the infant weighed less than 2∙5kg at birth.

§Observations used = 3571; –2LL = 107 170; Wald *P* < 0.0001.

||Observations used = 3571; –2LL = 101 093; Wald *P* < 0.0001.

¶Observations used = 3571; –2LL = 101 775; Wald *P* < 0.0001.

**Observations used = 3571; –2LL = 95 350.75; Wald *P* < 0.0001.

††Self–reported databased on mother’s last CD4 cell count.

‡‡Significant relationship between characteristic and MTCT.

**Table 4** demonstrates the protective effect of advanced antiretroviral regimens on early MTCT within exclusively breastfeeding populations (2.17% [1.23–3.09%]) in the EBF group with advanced regimens compared with 12.17% (4.7–19.6%) in the no ARV group. Among EBF women who initiated cART in the first trimester or before, early MTCT was 0.82% (0.06–1.58%), data not shown in the table.

**Table 4:** Stratified analysis of MTCT by infant feeding practice and PMTCT regimen

Feeding pattern over previous 8 d at 6 weeks	Characteristic	Advanced regimen group*, *P* = 0.27	Other ARV regimen group*, *P* = 0.44	No known ARV group*, *P* = 0.05
EFF	Unweighted number–**U**WNo. HEI (weighted number WNo HEI)	1770 (234 655)	235 (30 524)	210 (25 226)
	**U**WNo HIV positive infants–HPI (WNo HPI)	29 (3383)	7 (739)	10 (1097)
	Weighted MTCT risk % (95% CI)	1.44 (0.92–1.97)	2.40 (0.53–4.31)	4.35 (1.39–7.30)
EBF	**U**WNo. HIV–exposed infants (HEI) (WNo HEI)	1166 (1554)	196 (25 414)	97 (12 775)
	**U**WNo HPI (WNo HPI)	25 (3374)	6 (1115)	10 (1555)
	Weighted MTCT risk % (95% CI)	2.17 (1.25–3.09)	4.39 (0.57–8.21)	12.17 (4.7–19.6)
MBF	**U**WNo. HEI (WNo HEI)	227 (29 465)	53 (6171)	93 (11 926)
	**U**WNo HPI (WNo HPI)	3 (329)	3 (326)	6 (726)
	Weighted MTCT risk % (95% CI)	1.12 (0.0–2.46)	5.27 (0–11.04)	6.08 (1.04–11.12)

MTCT – mother–to–child transmission, PMTCT – prevent mother–to–child transmission, EFF – formula feeding (no breastmilk), EBF – exclusive breastfeeding, MBF – breastfeeding with other fluids or solids, **U**WNo – unweighted number, WNo – weighted number; HEI – HIV–exposed infants, HPI – HIV positive infants, CI – confidence interval

*As defined in **Figure 1****.**

## DISCUSSION

These PMTCT surveillance studies conducted at national and subnational (provincial) levels, in public health facilities that provide care for the majority of South Africa’s children, demonstrated that population–level early MTCT was sustained at 2.6%, 17–24 and 31–38 months after PMTCT Option A policy was adopted. HIV testing uptake was 95% and at least 99.7% of tested mothers received their HIV test results; thus almost 95% of all mothers receive their HIV test results. Therefore the ≥95% validation target for HIV testing uptake has just about been met in 2012–13. Maternal antiretroviral uptake among mothers with EIA positive infants was 91.3% (90.0–92.6%) in the 2012–13 survey, also meeting this EMTCT validation target at national level.

Although the 2.6% was a reduction from previous MTCT estimates in South Africa, these data show that the national target of <2% at 6 weeks postpartum was not achieved. Uptake of first trimester cART reduced early MTCT to 1.17% (0.61–1.72). Extrapolating these results to numbers, assuming 32% infant HIV exposure among 100 000 live births, 2.6% early MTCT means that by 6 weeks postpartum, 832 infants per 100 000 live births were HIV infected, and 1.17% early MTCT means that 384 infants per 100 000 live births were HIV infected. These extrapolations are far higher than the EMTCT target of ≤50 HIV infected infants per 100 000 live births, and are driven by the high HIV prevalence in South Africa.; These stark findings illustrate the importance of a public health approach to PMTCT, which locates all PMTCT interventions within a comprehensive framework aiming to reduce new HIV infections among young women of reproductive age, thus reducing antenatal HIV prevalence. Although the paper confirms that early cART initiation is feasible and critical for further reducing MTCT in high HIV prevalence settings, it substantiates the view that prong 3 can reduce but not eliminate MTCT.

Advanced antiretroviral therapy (any cART or ARVP to mother before the third trimester, with infant prophylaxis) compared to no PMTCT drug interventions, reduced the MTCT associated with exclusive breastfeeding (EBF) from 12.17% to 2.17% (1.25–3.09%), and first trimester cART reduced EBF–associated MTCT to 0.82%.

The overall MTCT risk declined by 22% from the 3.5% (95% CI 2.9–4.2%) achieved under dual prophylaxis/2006 WHO PMTCT guidelines/2008 SA PMTCT guidelines [11]. Similarly any cART–use under PMTCT Option A policy demonstrated tendency toward lower early MTCT (Column B in **Table 2**, 1.6% [1.15–2.05%]) compared with cART–use reported in the 2010 survey [11], under 2008 SA PMTCT guidelines) (2.1% [95% CI 1.2–3.0%]), illustrating the population–level effect of cART initiation at higher CD4 cell counts. The results confirm that PMTCT impact can be sustained at national level, despite increasing breastfeeding uptake. The increased breastfeeding between 2011–12 and 2012–13 could be explained by the August 2011 national policy change (South African Tshwane Declaration of Support for Breastfeeding) to supporting breastfeeding among all women, regardless of HIV status. Formula feeding was only supported in special circumstances and for medical indications. The phasing out of free commercial infant formula for HIV positive women, as part of the PMTCT program began in January 2012 and free formula was fully withdrawn by September 2012.

This was the first national evaluation of PMTCT Option A in a breastfeeding setting. Interestingly, the two population–level surveys yield lower MTCT estimates compared with recent clinical trials conducted in breastfeeding settings [7]. Short–course maternal cART or long–course maternal ARVP with infant extended or short–term prophylaxis reduced early MTCT to 3.3%, 4.5% or 6.5%. The former result is from the Kesho Bora study which offered cART from 28–36 weeks gestation till 6 months postpartum to women with CD4 cell counts between 200 and 500 cells/mm^3^ [19]. The latter two results are from the Breastfeeding Antiretroviral and Nutrition (BAN) study which provided daily infant NVP or maternal cART from 1 week till 6 months postpartum, respectively [20].

Also of note is that these population–based findings do not corroborate modeling estimates, which suggest that reducing MTCT to <2% at 6 weeks and <5% at the final endpoint can only be achieved with >90% coverage with ART, greater than 50% reduction in incident HIV infection and 0% unmet need for family planning [21]. In the pooled analysis MTCT under PMTCT Option A was <2% among women who received cART before or during the first trimester (1.17%, 0.6–1.7%). However our calculations demonstrate that number of HIV infected children remain unacceptably high, being driven by high maternal HIV prevalence.

Among “middle–income” countries EMTCT has only been reported from Cuba, a non–priority country for MTCT elimination, where adult and antenatal HIV prevalence is very low (<0.1%) and a comprehensive national PMTCT program began in 1986, transitioning to PMTCT Option B+ in 2011 [22].

The results of our national surveys were limited by low provincial sample realization, which was addressed by weighting the data; exclusion of sick or dead infants, which may have over–estimated PMTCT effectiveness; lack of initial and current CD4 cell count and viral load data which precluded more in–depth analysis of MTCT by viral load or CD4 cell count, use of self–reported data on ARV use, and few events (outcomes) within antiretroviral regimen groups which reduced precision. Notwithstanding these, consistency between the PMTCT survey’s self–reported HIV sero–prevalence (29.5%) and anonymous annual antenatal survey’s HIV sero–prevalence (29.5%), despite the use of different sampling frames, confirms the robustness of the self–reported data.

## CONCLUSIONS

Three years after changing to PMTCT Option A, a sustained lower risk of early MTCT was measured at population level. Despite the existence of a mature PMTCT program with increasing cART coverage, there were still missed opportunities for PMTCT interventions particularly among undiagnosed HV positive mothers. MTCT was reduced to less than 2% only among mothers who initiated cART during the first trimester or before. Despite a reduction in percentage MTCT, the number of infant HIV infections per 100 000 live births at six weeks postpartum, was above the global validation target. Eliminating unidentified maternal HIV infections [23,24], reducing maternal HIV prevalence and improving retention in HIV–related care (early cART initiation and adherence) [25] are critical to closing current gaps.

These periodic surveys, conducted nationally among all children attending public health facilities for immunization regardless of their mothers HIV status, have been pivotal in tracking national and subnational PMTCT impact in South Africa. While routine systems are being strengthened to monitor PMTCT impact, national surveys, such as these reported in this paper, conducted every two to three years are key for tracking PMTCT impact. Where routine systems are strong, periodic surveys conducted every four to five years may be important to validate routine data.
